# Effect of *Stenotrophomonas maltophilia* on Tuberculosis

**DOI:** 10.1128/spectrum.00944-23

**Published:** 2023-06-12

**Authors:** Yue Li, Ailan Zhao, Qin Yu, Nan Yu, Yao Cui, Xiaohan Ma, Haican Liu, Ruibai Wang

**Affiliations:** a State Key Laboratory for Infectious Disease Prevention and Control, National Institute for Communicable Disease Control and Prevention, Chinese Centre for Disease Control and Prevention, Beijing, China; b Tuberculosis Clinic, Chaoyang District Centre for Disease Control and Prevention, Beijing, China; Huashan Hospital of Fudan University

**Keywords:** tuberculosis, *Stenotrophomonas maltophilia*, co-infection, multidrug-resistant, decay

## Abstract

Tuberculosis (TB) is an important infectious disease suffered by many countries, including China. In this stage, accurate diagnosis and treatment are key measures for the prevention and control of TB. Stenotrophomonas maltophilia is a global emerging Gram-negative, multidrug-resistant (MDR) organism characterized by its high contribution to the increase in crude mortality rates. By single cell preparation and strain identification, we isolated S. maltophilia from stored cultures of Mycobacterium tuberculosis (Mtb). We found that S. maltophilia could not be removed from sputum by alkali treatment or inhibited by antibiotic mixture added to MGIT 960 indicator tubes. When co-cultured with Mtb on a Löwenstein-Jensen (L-J) slant, it could inhibit the growth of Mtb and liquefy the medium. More seriously, it was resistant to 10 of the 12 anti-TB drugs, including isoniazid and rifampin, and made the mixed samples display multidrug-resistant Mtb (MDR-TB) results in the drug sensitivity test, which might change a treatment regimen and increase disease burden. Following, we conducted a small-scale surveillance which showed that the isolation rate of S. maltophilia in TB patients was 6.74%, but these patients had no special characteristics and the presence of S. maltophilia was hidden. The effect of *S. maltophilus* on TB and its mechanism are unclear and require more attention.

**IMPORTANCE** China is a high-burden country for tuberculosis (TB), multidrug-resistant/rifampicin-resistant tuberculosis (MDR/RR-TB), and HIV-associated TB. Increasing the positive rate of culture and the accuracy of antibiotic susceptibility testing (AST) are important for diagnosis, treatment, and control of TB. In our study, we found that the isolation rate of Stenotrophomonas maltophilia in TB patients was not neglectable and that this bacterium affects the isolation and AST results of TB. Due to a lack of relevant research, the impact of S. maltophilia on the course and outcome of TB is unclear. However, the characteristics of S. maltophilia that increase disease mortality require attention. Therefore, in the clinical testing of TB, in addition to mycobacteria, it is recommended to increase the detection of co-infected bacteria and improve the awareness of TB clinicians of these bacteria.

## INTRODUCTION

Tuberculosis (TB) is an important infectious disease suffered by many countries, including China. In 2021, an estimated 6.4 million people were newly infected with TB worldwide, with an estimated 1.6 million deaths (1). In 2015, the World Health Organization announced the End TB Strategy, which called for a 90% reduction in TB deaths and an 80% reduction in the TB incidence rate by 2031 compared with that in 2015. In this stage, accurate diagnosis and treatment are key measures to achieve the targets set by this strategy. However, in 2020, only 59% of the 4.8 million pulmonary TB patients were confirmed by bacteriology (1). There is a big gap between the positive rate of culture and the diagnostic rate.

Stenotrophomonas maltophilia is a new global emerging Gram-negative, multidrug-resistant (MDR) organism (MDRO) (2), and one of the most common intensive care unit (ICU)-acquired and nosocomial infection strains (3–5). Data from the China Antimicrobial Surveillance Network (CHINET) showed that S. maltophilia ranked 5th to 6th among all Gram-negative bacteria and 3rd among nonfermentative bacteria, accounting for 3.5% to 7.1% of all clinical isolates from 2005 to 2022. The most attractive aspects of this Gram-negative bacterium are its high contribution to the increase in crude mortality rates (2, 6), its resistance to various classes of antimicrobials (7, 8) and its ability to cause various serious infections in susceptible patients (6, 9), although S. maltophilia is not a highly virulent pathogen and is not associated with worse clinical outcomes *per se*. The mortality rates of bloodstream infection and ventilator-associated pneumonia caused by this bacterium are 14% to 69% and 10% to 30%, respectively. Even in non-neutrophil-deficient and non-ICU patients, the attributable mortality of S. maltophilia lung infection is as high as 20% to 30% (10). For S. maltophilia, the main relevant high-risk patient groups are cystic fibrosis (CF), chronic obstructive pulmonary disease, and cancer, particularly obstructive lung cancer.

S. maltophilia has been co-isolated with several other microorganisms in patient samples (11). Although Mycobacterium tuberculosis (Mtb) is the world’s most threatening bacterial pathogen and TB is a typical chronic respiratory disease with far more patients than CF, TB patients were not included in the concerned population infected with S. maltophilia. We speculated that the main reason for this is that the isolation method of Mtb is completely different from those of other respiratory pathogens. Although surveillance of S. maltophilia infections has been conducted in hospitals in several different countries (12–14), there have been only a few reports of co-infection/co-isolation of Mycobacterium with S. maltophilia, besides two case reports of M. chelonae and M. kansasii (15, 16). When testing the purity of stored Mtb strains, we isolated a S. maltophilia strain. To clarify the status of S. maltophilia co-infection/co-isolation among TB patients, we conducted a small-scale surveillance in a district TB hospital in Beijing.

## RESULTS

### *S. maltophilia* isolation and its growth on media.

**FIG 1:**
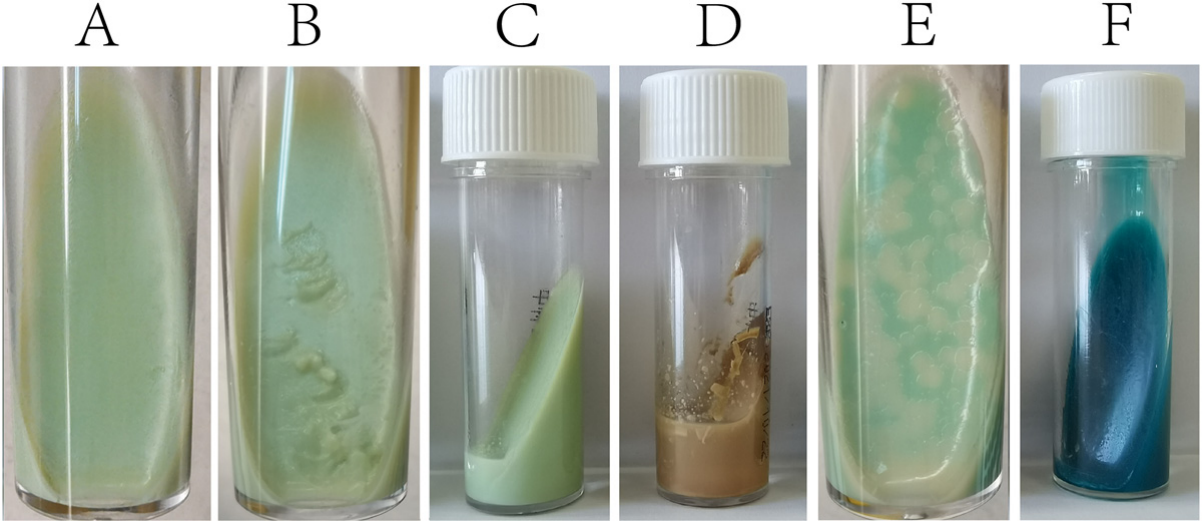
L-J slants after inoculation. (A and B) L-J slant inoculated with S. maltophilia strain 11066 within 48 h had no noticeable change, but the surface was actually softened. (C) L-J slant inoculated with S. maltophilia after 10 days incubation, with the liquid observable at the bottom of the tube. (D) L-J slant inoculated with S. maltophilia after approximately 1 month. (E) Flat, translucent white colonies of B. subtilis grown on an L-J slant. (F) L-J slant, with the egg component replaced with 10% OADC and 1.5% agar, inoculated with S. maltophilia.

We identified S. maltophilia strain 11066 from a smooth and transparent clone on 7H10 + 10% oleic acid-albumin-dextrose-catalase (OADC) plates which were inoculated with the filtered supernatant of stored Mtb cultures and incubated overnight. Alkali treatment with NaOH-NALC (*N*-acetyl-1-cysteine) for 15 to 20 min cannot kill S. maltophilia. S. maltophilia growth on L-J slant does not form obvious colonies, so it is more likely to be judged as culture-negative. When 100 μL of a 1.0-McFarland suspension of S. maltophilia strain 11066 was inoculated on an L-J slant, the surface of the slant was softened within 48 h without noticeable changes (Fig. 1A and B). After 10 days of incubation, liquid had clearly accumulated at the bottom of the tube (Fig. 1C). When the incubation time exceeded 1 month, the slant turned brown and almost completely collapsed (Fig. 1D). Bacillus subtilis is a common laboratory-contaminating strain that, like S. maltophilia, cannot be killed by alkaline treatment, can grow on L-J slant, and causes slight surface softening. In addition, there are literature reports on the lysis effect of B. subtilis on Mtb (17). Therefore, in this experiment, we used B. subtilis 2216 as the growth control. However, unlike S. maltophilia, B. subtilis could form flat, translucent white colonies on the L-J slants (Fig. 1E) which could be easily distinguished from Mtb by their colony morphology.

The pH of the liquefied L-J medium was between 6.67 and 7.06, a slight reduction from the pH range of 6.8 to 8.0 of uncultured L-J medium. Modified L-J medium with 10% OADC and 1.5% agar replacing the egg component was neither softened nor liquefied when inoculated with S. maltophilia 11066 (Fig. 1F), suggesting that the degradation of the egg components is responsible for the observed degradation of the slant.

Cultures of S. maltophilia 11066 at 0.5 McFarland were 10-fold serially diluted eight times, and 500 μL of each dilution was inoculated into a MGIT culture tube with growth supplement and PANTA (polymyxin B, amphotericin B, nalidixic acid, trimethoprim, and azlocillin) and cultured in Bactec MGT 960. Six of the nine culture tubes were reported as positive at 3, 5, 6, 8, 15, and 26 days, respectively. The remaining three tubes with inoculation concentrations of less than 10^2^ CFU/mL were reported as negative over 42 days. The same assay performed with B. subtilis strain 2216 were negative at all concentrations and time points, indicating that PANTA could inhibit the growth of B. subtilis but had no inhibitory effect on S. maltophilia.

### Mixed cultures of Mtb with *S. maltophilia* or *B. subtilis* grown on L-J slants.

**FIG 2:**
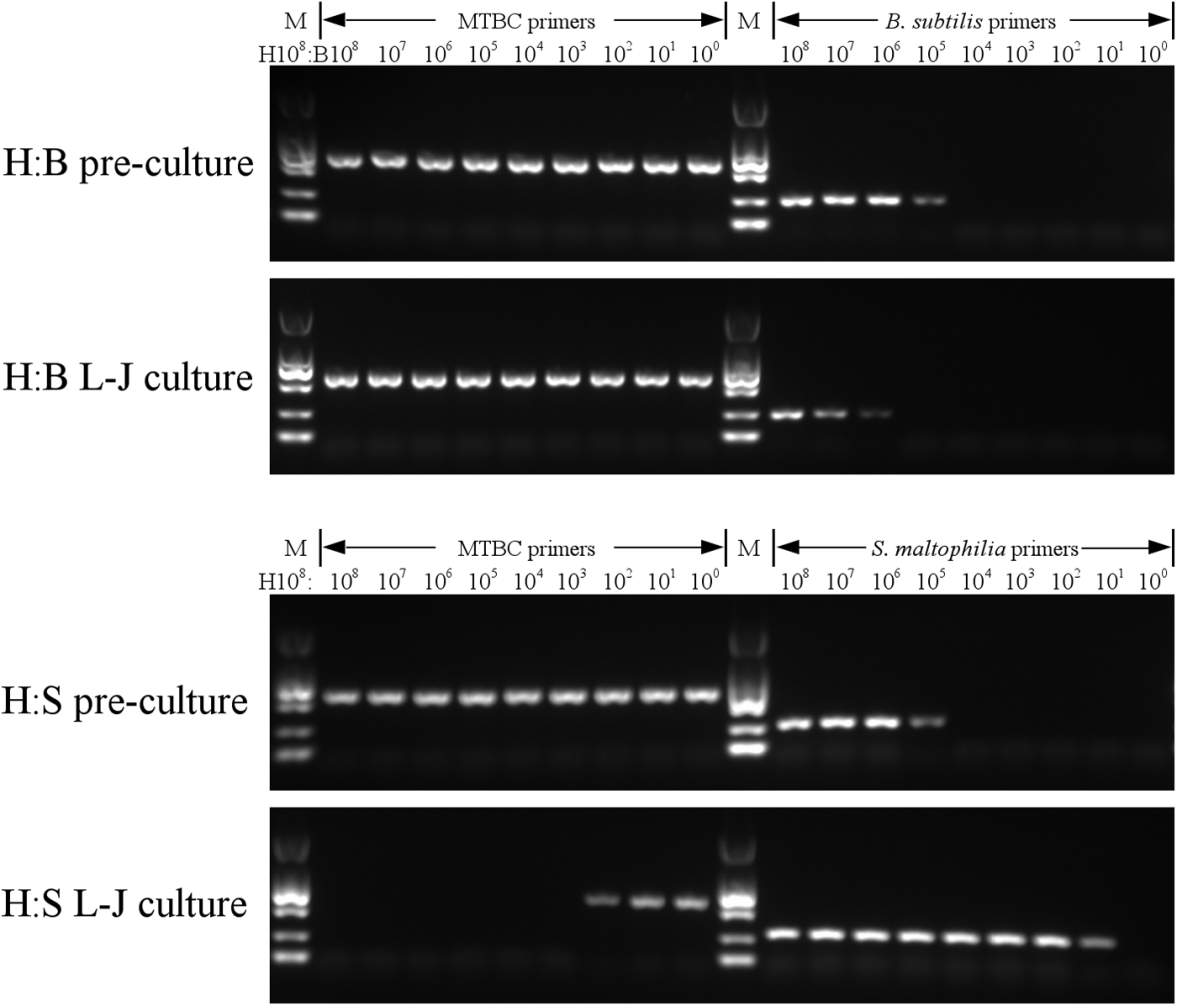
PCR amplification results. A mixed culture of 10^8^ CFU/mL Mtb reference strain H37Rv with nine serial dilution concentrations of either B. subtilis or S. maltophilia on L-J slants.

The surface of the L-J slants inoculated with mixed Mtb and a concentration of S. maltophilia between 10^8^ to 10^4^ CFU/mL softened after 10 days of incubation, and liquid was present at the bottom of the tubes. In these tubes, Mtb was not detectable by PCR after incubation. In the last four tubes inoculated with S. maltophilia concentrations of less than 10^3^ CFU/mL, some surface areas were not softened and Mtb was detectable by PCR. Meanwhile, S. maltophilia was detectable by PCR in all tubes after incubation, including the five tubes that were initially below the detection limit (Fig. 2). In the same experiment measuring growth competition between Mtb and B. subtilis, only the two tubes inoculated with 10^8^ and 10^7^ CFU/mL B. subtilis had partially softened surfaces after incubation. The detection results of Mtb and B. subtilis by PCR were identical before and after incubation. These results indicate that S. maltophilia outcompeted Mtb when they were cocultured on L-J slants, while B. subtilis appeared to coexist with Mtb. Only in tubes where the ratio of Mtb to S. maltophilia was greater than 1 × 10^5^:1 could Mtb be detected by PCR after incubation.

### Small-scale surveillance in tuberculosis clinic.

Sputum samples were collected from 89 pulmonary TB patients. All the colonies with different morphologies grown from untreated sputum on the 7H10 + 10% OADC plate were identified by 16S rRNA gene sequencing. Among them, the strains with more than 90% identity with S. maltophilia in sequence blasting were further amplified and sequenced with seven primer pairs in the MSLT scheme of S. maltophilia. Six S. maltophilia strains were isolated, together with one Stenotrophomonas rhizophila strain, one Stenotrophomonas terrae strain, and one strain which had the highest identity to *Stenotrophomonas* sp. strain ZAC14D2_ MKIMI4 without certain species. The samples of these patients from further consultations had identical test results. The isolation rate of S. maltophilia in this study was 6.74%.

The six S. maltophilia-positive patients included an 88-year-old male and a 60-year-old male, and the remaining two males and two females were between 24 and 38 years old. Two patients were initially treated and four were retreated. Three of the six patients were positive for sputum smear or Mtb culture. Only one patient had a white blood cell count within the normal range, and the remaining patients were below the normal value. All patients had no other abnormal symptoms except for cough and expectoration of TB. No cephalosporin antibiotics had been used for treatment during the early stage, and no invasive surgical treatment had been performed.

### MLST typing.

**FIG 3:**
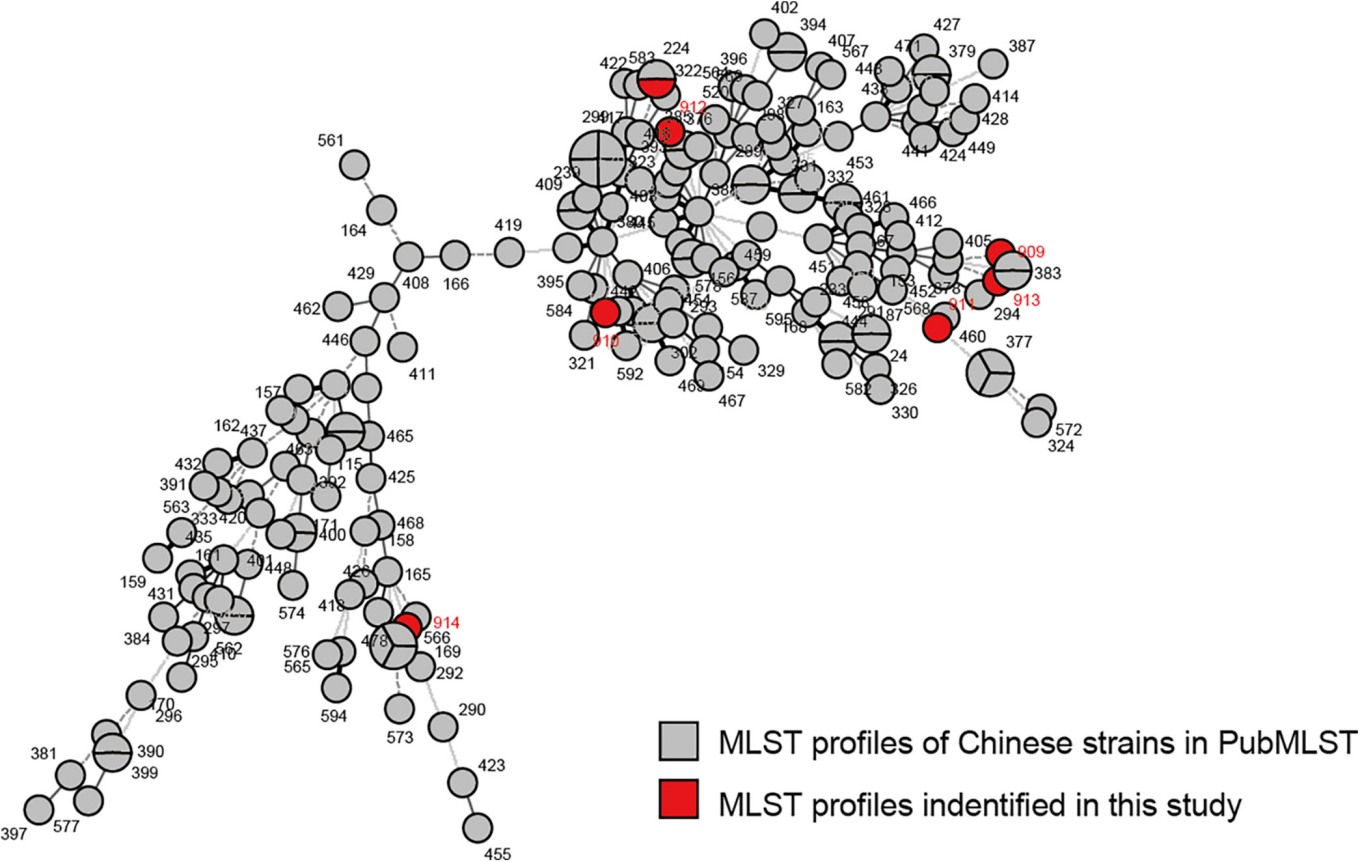
MLST cluster tree of strains isolated in China in the PubMLST database, drawn by BioNumerics (version 7.6.3). Gray circle dots are MLST profiles of Chinese strains, and the seven red dots are MLST profiles of the strains identified in this study.

All of the seven S. maltophilia strains isolated in this study were submitted to PubMLST and received IDs from 1173 to 1179 (Table 2). Among these, only strain 435A1 had a multilocus sequence typing (MLST) allelic profile previously included in PubMLST, namely, ST_224. From the cluster tree, we could see that the strains isolated this time had no aggregation and were scattered in the sequence types (STs) isolated in China (Fig. 3). This is consistent with previous findings that S. maltophilia had considerable heterogeneity and that clinical isolates had a higher mutation rate than environmental isolates for adapting to the environment within different areas of patient lungs (2, 18, 19).

**TABLE 1:** The primers used in this study^*a*^

Primer	Sequence	Note
16S-U	AGA GTT TGA TCM TGG CTC AG	Universal primers for bacteria
16S-L	CCG TCA ATT CMT TTR AGT TT	
ku-MTBC-U	GGT GGT CGA CTA CCG CGA TCTT	MTBC specific
ku-MTBC-L	TCT TCG GGC TCG TCC AGC AAC C	
SM 1	GCT GGA TTG GTT CTA GGA AAA CGC	*S. maltophilia* specific
SM 2	ACG CAG TCA CTC CTT GCG-3	
SM 3	CAG CCT GCG AAA AGT A	
SM 4	TTA AGC TTG CCA CGA ACA G	
Sme1-F	GCA TGA TCT CCA TSG TYT TG	
Sme1-R	GGC ACT TCA AGA ACA AGA GC	
B.su-gyrA-F	TCT GCT CGT GAA CGG TGC T	*B. subtilis* specific
B.su-gyrA-R	CTC AGC TTT TGC CCG GAT C	

a MTBC, Mycobacterium tuberculosis complex.

**TABLE 2:** The 7 allelic genes and MLST profiles of S. maltophilia strains identified in this study

Strain			Allelic gene							ST
No.	ID	Name	*atpD*	*gapA*	*guaA*	*mutM*	*nuoD*	*ppsA*	*recA*	
1	1173	517A1	199^*a*^	8	350	211	18	200	201	909^*a*^
2	1174	598A	110	259^*a*^	549^*a*^	264^*a*^	206^*a*^	289^*a*^	251^*a*^	910^*a*^
3	1175	580A	150	173	551^*a*^	265^*a*^	4	200	148	911^*a*^
4	1176	435A1	93	100	93	59	63	69	94	224
5	1177	546A3	77	85	548^*a*^	100	81	94	75	912^*a*^
6	1178	678A	200^*a*^	260^*a*^	550^*a*^	33	205^*a*^	200	158	913^*a*^
7	1179	11066	83	4	326	46	108	83	133	914^*a*^

a New allelic genes and MLST profiles identified in this study.

### Antibiotic resistance of *S. maltophilia*.

The AST results of the seven S. maltophilia strains were similar. They were only sensitive to 2 of the 12 tested anti-TB drugs: ofloxacin and moxifloxacin. They were resistant to rifampin, amikacin, rifabutin, cycloserine, and kanamycin when judged by the recommended breakpoint concentrations for Mtb. The MICs of the remaining five drugs, streptomycin, para-aminosalicylic acid, ethionamide, isoniazid, and ethambutol, exceeded the maximum detection concentrations of the assay. These results showed that the S. maltophilia strains were extremely resistant to anti-TB drugs. The effect of S. maltophilia on the AST result of Mtb depended on the ratio of these two strains in the tested samples. When the rate of *S. maltophilus* is high, fewer days are required for the growth control to be reported as positive, and the result is easily judged as pollution. However, when the rate of *S. maltophilus* is low, the days for reporting positive results will not be changed much, and the AST result is more likely to be misreported as multidrug-resistant Mtb (MDR-TB) because S. maltophilia is resistant to isoniazid and rifampin (1).

Of the 29 drugs on the BD NMIC 413 panel, all of the S. maltophilia strains were sensitive to 9 drugs, chloramphenicol, fosfomycin (G6P), levofloxacin, minocycline, moxifloxacin, norfloxacin, tetracycline, tigecycline, and trimethoprim-sulfamethoxazole; and were resistant to 11 drugs, amikacin, aztreonam, cefazolin, cefoxitin, ceftriaxone, cefuroxime, ciprofloxacin, gentamicin, imipenem, nitrofurantoin, and tobramycin. Sensitivity to the remaining 9 drugs, amoxicillin-clavulanate, ampicillin-sulbactam, cefepime, cefoperazone-sulbactam, ceftazidime, colistin, ertapenem, meropenem, and piperacillin-tazobactam, varied among strains.

## DISCUSSION

Clinically, the isolation and cultivation of S. maltophilia and Mtb follow two completely different procedures. For most respiratory pathogens, including S. maltophilia, sputum samples or throat swabs are inoculated for at least 5 days on nonselective sheep blood agar and selective solid media such as chocolate agar with bacitracin, MacConkey agar (for Gram-negative bacteria), Columbia agar with colistin, and nalidixic acid agar (for Gram-positive bacteria), and Sabouraud Dextrose Agar with antibiotics (for fungi) (20). For Mtb, the sputum samples are pretreated with NALC-NaOH, which can kill most respiratory bacteria, and then inoculated on L-J slant or into MGIT culture tubes. L-J slant has an advantage over Mtb culture, but its limited surface area makes it difficult to obtain true pure cultures of Mtb and to detect mixed or contaminated samples unless the growth has an obvious abnormal appearance. From our research, we could see that neither alkali treatment nor the PANTA mixture could remove S. maltophilia from the sample. Its growth on the L-J slant is hidden, while its presence increases the false-negative isolation rate of Mtb. The more serious implication of this study is that the presence of S. maltophilia in an Mtb sample may result in a misdiagnosis of MDR-TB, which has a different treatment regimen from drug-susceptible Mtb and a higher financial cost. Therefore, it is necessary to strengthen the detection of S. maltophilia in TB samples. On the 7H10 + 10% OADC plate, S. maltophilia and Mtb, as well as other respiratory tract bacteria, are easily distinguished from each other by their growth rate and colony morphology. Although the 7H10 + 10% OADC plate is not highly selective, the number of colonies is not too high for them to be inseparable when untreated sputum samples are spread or streak-inoculated on it. If the inoculated plate is sealed in a plastic self-sealing bag, it can be stored or incubated for several months. Therefore, we suggest that the 7H10 + 10% OADC plate should be added to the testing process to enforce the conventional L-J slant used for TB samples.

Degradation and liquefication of the L-J slant is a commonly observed indicator of contamination. It has been speculated that this phenomenon is caused by acidification of the L-J medium, but to our knowledge this has not been thoroughly studied. In this study, we found that both S. maltophilia and B. subtilis can cause softening of the L-J slant, but the liquefaction rate of medium inoculated with S. maltophilia is much higher than that of B. subtilis-inoculated medium, and the amount of liquid accumulated at the bottom is also much more obvious than that in B. subtilis. S. maltophilia is efficient at degrading keratin and its keratinase can act on different keratin sources, including feathers, hair, wool, and horns (21, 22). Therefore, it can be understood that S. maltophilia can also easily degrade the egg component in L-J slant, leading to the softening of the medium and the observed liquification of the slant.

The S. maltophilia genome encodes many types of extracellular enzymes (2). On the one hand, it can inhibit other bacteria. For example, its serine protease has activity against free-living and plant-parasitic nematodes. Two R-type phage tail-like bacteriocins, maltocin P28 (23) and S16 (24), and the modular bacteriocins stenocins (25), have antibacterial activity against Pseudomonas, Staphylococcus, Escherichia coli, etc. It is not clear whether the S. maltophilia factors which inhibit Mtb are among these known bacteriostatic agents or whether the Mtb inhibition and egg component degradation occur via the same mechanism.

On the other hand, S. maltophilia demonstrates cytotoxicity against host cells, with intensive rounding, loss of intercellular junctions, vigorous endocytosis, cell aggregation, and even death (26). Its elastase production can exacerbate influenza A virus infection (2). S. maltophilia can impair lung function in CF patients, leading to a lower mean percent predicted FEV_1_ (forced expiratory volume in 1 s) than that in patients never infected with S. maltophilia (2, 27, 28), which is why chronic S. maltophilia infection is listed as an independent risk factor for pulmonary exacerbation requiring hospitalization and antibiotic therapy (6). Although the impact of S. maltophilia on the course and outcome of TB has not been researched and more clinical observation is needed, it is noteworthy that except for deterioration of lung function, no common risk factors associated with S. maltophilia infection were observed in this study, such as old age, invasive procedures, antibiotic use, and ICU stay. It is more difficult to determine the presence of S. maltophilia in TB patients without targeted culture and detection.

In conclusion, the culture-based isolation rate of S. maltophilia in TB patients indicates that it is a major co-occurring species with Mtb and may be an important opportunistic pathogen for TB patients, like in CF patients. For clinicians, especially those engaged in tuberculosis prevention and treatment, more recognition and attention should be paid to this bacterium.

## MATERIALS AND METHODS

### Sample collection.

Clinical Mtb-positive cultures were stored in the Mycobacterium culture collection at the National Institute for Communicable Disease Control and Prevention. Small-scale surveillance was conducted at the tuberculosis clinic at the Chaoyang District Centre for Disease Control and Prevention from July to October 2022, under approval from the Ethics Committee of the National Institute for Communicable Disease Control and Prevention (no. ICDC-2022010), and written informed consent was obtained from participants. This tuberculosis clinic is the only TB-designated hospital in Chaoyang District which receives and cures suspected or confirmed TB patients from more than 100 hospitals in Chaoyang District, which has a population of 3.452 million.

### Strain isolation and culture.

First, 50 μL sputum was inoculated onto 7H10 + 10% OADC agar plates. Next, the sputum sample was subjected to alkaline decontamination treatment with NALC and 4% NaOH for 15 min, and neutralized with phosphate buffer (pH 6.8). After centrifugation, a resuspended 100-μL portion was inoculated on the L-J slant. MGIT 960 indicator tubes (MGIT) (Becton, Dickinson, Sparks, MD, USA) supplemented with OADC and PANTA antibiotic mixture were used to test the growth of S. maltophilia. The pH of the liquid in the degraded L-J slants was measured by a Seven2Go portable pH meter with an InLab Ultra-Micro-ISM electrode (Mettler-Toledo GmbH, Shanghai, China).

### Purity test of stored Mtb cultures.

Mtb often forms multicellular aggregates that are very difficult to disperse (29). Commonly used dispersal methods include glass bead grinding and sonication; however, these methods cannot guarantee that the suspension obtained after treatment is completely single-cell, which may hinder the isolation and identification of strains in mixed samples. Mtb measures 1 to 4 × 0.4 μm in size. Therefore, the stored Mtb cultures were treated as follows: 100 μL of bacterial solution, stored frozen, was added into 1 mL saline. After dispersion by sonication with a BACspreader 1100 (TB Healthcare Group, Guangdong, China), the bacterial supernatant was filtered through a 0.65-μm polyvinylidene difluoride membrane (DVPP02500, Merck Millipore Ltd., Ireland) to remove any remaining aggregates. A loop of the filtered supernatant was then streaked on 7H10 + 10% OADC plates for single colonies.

### Strain identification and typing.

The clones were identified by amplification with the 16S rRNA gene universal primers for bacteria (16S-U/L) and sequencing. Three S. maltophilia-specific primer sets (30–32), one B. subtilis-specific primer set, and Mtb-specific primers (33) were used in the following detections (Table 1). The MLST scheme (34), primers, MLST profiles, and allele sequences of S. maltophilia were obtained from the PubMLST database (35).

### Antibiotic susceptibility testing.

The Sensititre MYCOTB MIC plate (Trek Diagnostic Systems, Cleveland, OH, USA) was used to detect the sensitivity of strains to 12 anti-TB drugs, and BD Phoenix NMIC 413 panels on BD Phoenix 100 equipment (BD, Sparks, MD, USA) was used to determine their sensitivity to 29 drugs commonly used for Gram-negative strains. The MICs were determined according to Clinical and Laboratory Standard Institute (CLSI) standards (36).

### Co-culture of Mtb with *S. maltophilia* and *B. subtilis* on L-J slants.

Bacterial suspensions of 1.0 McFarland (approximately 3 × 10^8^ CFU/mL) of Mtb strain H37Rv, S. maltophilia strain 11066, and B. subtilis strain 2216 were prepared. Next, suspensions of S. maltophilia and B. subtilis were serially diluted 10-fold eight times (from 10^7^ to 10^0^ CFU/mL). For each dilution concentration, a 100-μL aliquot was mixed with a 100-μL aliquot of H37Rv (10^8^ CFU/mL). Half of the mixture (100 μL) was used to inoculate L-J slants, which were cultured at 37°C for 10 days. The slants were then washed with 1 mL saline, and the presence of the bacterial strains in the washing solution was confirmed via PCR.

### Data availability.

New MLST allelic gene sequences and profiles identified in this study have been submitted to PubMLST (https://pubmlst.org/organisms/stenotrophomonas-maltophilia), including *atpD* (atpD_199 ~ atpD_200), *gapA* (gapA_259 ~ gapA_260), *guaA* (guaA_549 ~ guaA_550), *mutM* (mutM_264 ~ mutM_265), *nuoD* (nuoD_205 ~ nouD_206), *ppsA* (ppsA_289), *recA* (recA_251), and profile (ST_909 ~ ST_914).
